# In Memoriam—Dr. Mary Bates Stevens

**Published:** 1897-05

**Authors:** 


### IN MEMORIAM—DR. MARY BATES STEVENS.

It is with great regret that we announce the death of the late Dr. Mary Bates Stevens, one of the oldest, and one of the most faithful subscribers to THE HOMEOPATHIC PHYSICIAN.

She died February 15th, 1897, at Clover Hill Farm, South Turner, Maine.

Dr. Stevens was born March 18th, 1839.

She graduated from the New York Medical College and Hospital for Women in 1875.

She was an exceptionally rare woman; a student of constant and untiring industry, and was well known and acknowledged to be one of the best read physicians of the school; none exceeding her in her professional attainments. She was thoroughly devoted to her profession, and had a large and successful practice in the city of Auburn, Maine, for many years.

She was a strong and consistent adherent of Homeopathy, and practiced it unfailingly and without wavering to the end.

Her life was graced with kind and charitable acts, and she was a leader and worker in many benevolent organizations. None so poor and down-trodden but could have the best of her skill and care.

Naturally not blessed with physical strength, yet she was enabled to accomplish a great amount of work in the several lines in which she was so deeply interested, by the sustaining power of a strong will.

She leaves a husband to mourn her loss.

